# The Association of Intravenous Iron Administered the Day before Total Knee Arthroplasty with Postoperative Anemia and Functional Recovery

**DOI:** 10.3390/medicina59071212

**Published:** 2023-06-28

**Authors:** Ho Jung Jung, Min Wook Kang, Jong Hwa Lee, Joon Kyu Lee, Joong Il Kim

**Affiliations:** 1 Department of Orthopaedic Surgery, Chuncheon Sacred Heart Hospital, Hallym University College of Medicine, 77, Sakju-ro, Chuncheon-si 24253, Republic of Korea; 2 Department of Orthopaedic Surgery, Kangnam Sacred Heart Hospital, Hallym University College of Medicine, 1 Singil-ro, Yeongdeungpo-gu, Seoul 07441, Republic of Korea; 3 Department of Orthopaedic Surgery, Konkuk University Medical Center, Konkuk University School of Medcine, Seoul 05030, Republic of Korea

**Keywords:** iron, hemoglobin, anemia recovery, length of hospital stay, total knee arthroplasty

## Abstract

*Background and Objectives:* Total knee arthroplasty (TKA) involves blood loss, increasing the risk of postoperative anemia and delayed functional recovery. Intravenous (IV) iron supplementation limits postoperative anemia; however, the effectiveness of IV iron, administered one day before TKA, on postoperative anemia and functional recovery has scarcely been studied. *Materials and Methods:* We conducted a retrospective cohort study with propensity score matching using two consecutive groups of patients who underwent TKA using tranexamic acid: the iron group received 500 mg ferric derisomaltose intravenously one day before surgery (n = 46); the non-iron group did not (n = 46). Hemoglobin (Hb) level was determined at postoperative days (PODs) 2, 4, 6, 14, and 30. Ferritin, transferrin saturation (TSAT), and functional iron deficiency anemia (IDA) rate were measured at PODs 2, 4, 6, and 14. Length of hospital stay and transfusion rate were also evaluated. *Results:* The iron group had higher Hb levels at PODs 6, 14, and 30 and higher ferritin and TSAT at PODs 2, 4, 6, and 14. The functional IDA rate was significantly higher in the non-iron group at PODs 2, 4, 6, and 14. Length of hospital stay was significantly shorter in the iron group; however, the rate of transfusion did not differ between the two groups. *Conclusions:* IV iron administered one day before TKA was associated with postoperative anemia recovery and length of hospital stay; however, it did not lower the postoperative transfusion rate.

## 1. Introduction

Total knee arthroplasty (TKA) is associated with substantial blood loss and postoperative anemia, which may delay patient recovery, lengthen hospital stay, and eventually lead to poor clinical outcomes [1,2,3]. Additionally, patients with postoperative anemia may require allogenic red blood cell transfusion, potentially leading to transfusion-related complications [4,5]. Therefore, a patient blood management program is strongly recommended to optimize hemoglobin (Hb) levels during the perioperative period.

Oral or intravenous (IV) iron supplementation is a key element of patient blood management, with IV iron found to have a faster recovery effect on Hb levels and fewer side effects [6,7,8]. It is recommended that IV iron be administered for at least two to four weeks before TKA to optimize Hb levels [1]. However, this is not always possible, and it can be inconvenient for a patient to visit the outpatient clinic before surgery for IV iron supplementation. Moreover, several studies have reported that IV iron administration during the perioperative period assists postoperative anemia recovery [7,8,9]. Recently, Yoo et al. [10] and Park et al. [11] reported that intraoperative IV iron administration helped patients recover from postoperative anemia after TKA; however, during surgery, patients were hemodynamically unstable due to active bleeding. Additionally, various drugs are administered that may interact and potentially decrease the effectiveness of IV iron administration [12]. Thus, it is prudent to administer IV iron one day before surgery when the patient is hemodynamically stable and there is no risk of drug interactions. To the best of our knowledge, no study has analyzed postoperative anemia and functional recovery following IV iron administration one day before TKA.

Several factors can affect recovery from postoperative anemia, such as the total amount of iron as well as circulating iron levels [13,14]. However, the total amount of iron and circulating iron levels are not always proportional. Jeong et al. [4] analyzed changes in iron profiles after TKA and demonstrated that transferrin saturation (TSAT) levels can be low despite sufficient ferritin levels. After surgery, hepcidin quantities increase due to inflammation, resulting in decreased iron mobilization, which may lead to functional iron deficiency anemia (IDA) [15]. Indeed, Park et al. reported that functional IDA reached almost 50% on the fifth day after TKA [11]. Therefore, it is important to understand the incidence of functional IDA after TKA and evaluate whether the use of IV iron supplementation can reduce the occurrence of functional IDA. To date, no study has evaluated the difference in the occurrence of functional IDA after TKA depending on the perioperative use of IV iron.

We aimed to analyze the effect of IV iron administration one day before TKA on postoperative anemia and functional recovery and determine whether IV iron administration can reduce the occurrence of functional IDA. We hypothesized that IV iron administration one day before surgery would facilitate postoperative anemia and functional status recovery and could reduce the occurrence of functional IDA after TKA.

## 2. Materials and Methods

### 2.1. Patients

Following Institutional Review Board approval (IRB No. 2021-04-021), we performed a retrospective cohort study of 158 consecutive patients who: (1) underwent primary unilateral TKA for degenerative arthritis with Kellgren–Lawrence grade 3 or 4; and (2) either did or did not receive preoperative IV iron administration between April 2019 and November 2020. From February 2020, we administered preoperative IV iron to all patients undergoing TKA to improve Hb recovery. To avoid selection bias, consecutive patients received the same perioperative protocol with or without preoperative IV iron supplementation, depending on when the surgery was performed. The exclusion criteria were as follows: (1) presence of inflammatory arthritis, such as rheumatoid arthritis; (2) history of open knee surgery; (3) presence of other secondary arthritis conditions, such as infectious arthritis or traumatic destructive arthritis; and (4) incomplete medical records regarding iron panels and patient’s quality of life index. After the exclusion of 25 patients, a total of 87 patients were included in the non-iron group, and 46 patients were included in the iron group.

### 2.2. Surgical Intervention and Perioperative Management

In the iron group, one day before surgery, 500 mg ferric derisomaltose (Monofer^®^, Pharmacosmos A/S, Holbæk, Denmark) diluted with 200 mL of normal saline was administered intravenously over 2 h. Except for the IV iron administration, all patients underwent primary unilateral TKA according to the same surgical protocol. In both groups, a standard medial parapatellar approach was followed, with a tourniquet inflated to 300 mmHg. The surgical implant was a cemented posterior stabilized prosthesis in all patients. The tourniquet was deflated after the cement was completely set, and the prosthesis was firmly fixed. A suction drain was clamped and inserted before the capsule was closed, and a solution of 1 g tranexamic acid in 50 mL normal saline was injected into the articular space. The drain remained clamped for 2 h after the operation before being released.

Patients in both groups had the drain removed at 48 h postoperatively. From postoperative day (POD) 2 to POD 30, patients were administered aspirin as an anticoagulant. Range-of-motion exercises were started on POD 1, and walker ambulation began on POD 2. Transfusion of allogeneic blood was only indicated when the Hb concentration decreased below 8 g/dL with abnormal symptoms, such as tachycardia and hypotension.

### 2.3. Clinical Evaluations

We retrospectively collected preoperative data from medical records, including age, sex, body mass index (BMI), and American Society of Anesthesiologists Physical Status (ASA-PS) classification. The primary outcome variables were serial values of Hb and iron panels, including serum ferritin and TSAT, measured preoperatively and on PODs 2, 4, 6, 14, and 30. Using the values of iron panels and C-reactive protein (mg/L), rates of absolute and functional IDA were calculated preoperatively and on PODs 2, 4, 6, and 14. Absolute IDA was defined as low Hb (men < 13 g/dL, women < 12 g/dL) with TSAT < 20%, ferritin < 30 ng/mL, and no sign of inflammation; conversely, functional IDA was defined as low Hb (men < 13 g/dL, women < 12 g/dL) with TSAT < 20%, ferritin > 100 ng/mL, and CRP > 5 mg/dL [14,16].

The secondary outcome variables were transfusion rate and length of hospital stay (LOS). LOS was calculated as the number of days in the hospital from the day of surgery to the day of discharge. The primary target for LOS after TKA was set at six days in our institute. After that point, discharge was determined by agreement between the operator and physiotherapist that the patient was medically stable and that their functional ability was sufficient to allow discharge to the patient’s home. This included being able to climb up and down several steps, walk on flat ground using a walker for >10 min, and bend their operated knee for >90°. After discharge, patients were scheduled for a follow-up on PODs 14 and 30.

### 2.4. Statistical Analyses

**Figure 1:**
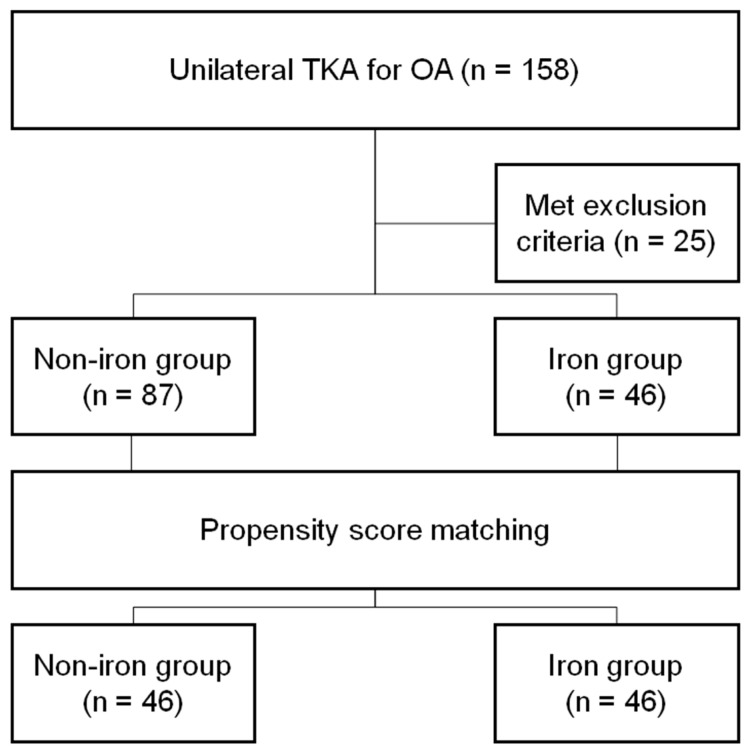
Flow diagram of the study. After propensity score matching, both groups comprised 46 patients. OA, osteoarthritis; TKA, total knee arthroplasty.

Before analysis, propensity score matching was performed using age, sex, BMI, and ASA-PS classification as variables. Propensity scores were generated using SPSS version 24 (IBM Corp., Armonk, NY, USA). Each patient in the iron group (n = 46) was assigned a matching patient in the non-iron group (n = 87), with a match tolerance (maximum difference between propensity scores) of 0.1. Therefore, 46 patients were ultimately included in each group (Figure 1). After matching, we performed independent t-tests for continuous data to compare the means and standard deviations. For binary data, percentages were compared using Pearson’s chi-square test and Fisher’s exact test. *p* < 0.05 was considered statistically significant. All statistical analyses were performed using SPSS version 24 (IBM Corp.).

## 3. Results

**Figure 2:**
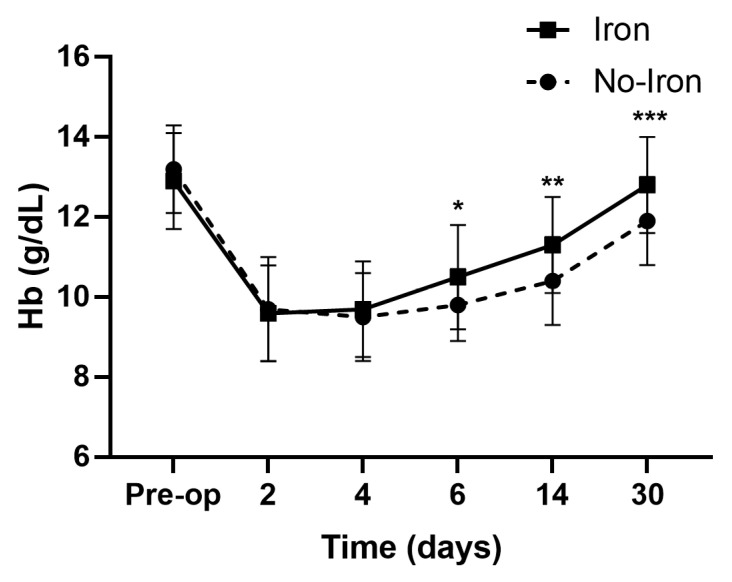
Serial changes in Hb. Hb values on postoperative days 6, 14, and 30 were significantly higher in the iron group than in the non-iron group. * *p* = 0.032, ** *p* = 0.021, *** *p* = 0.019. Hb, hemoglobin.

**Figure 3:**
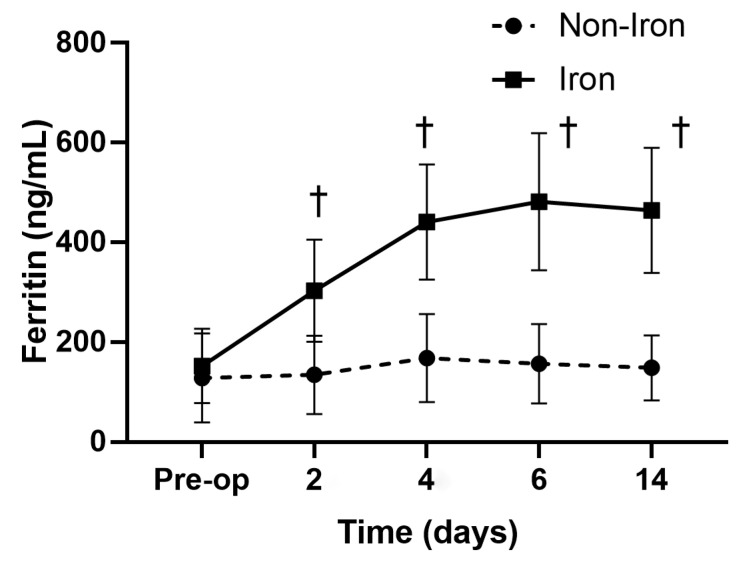
Serial changes in ferritin. Ferritin values on postoperative days 2, 4, 6, and 14 were significantly higher in the iron group than in the non-iron group. ^†^ *p* < 0.001.

**Figure 4:**
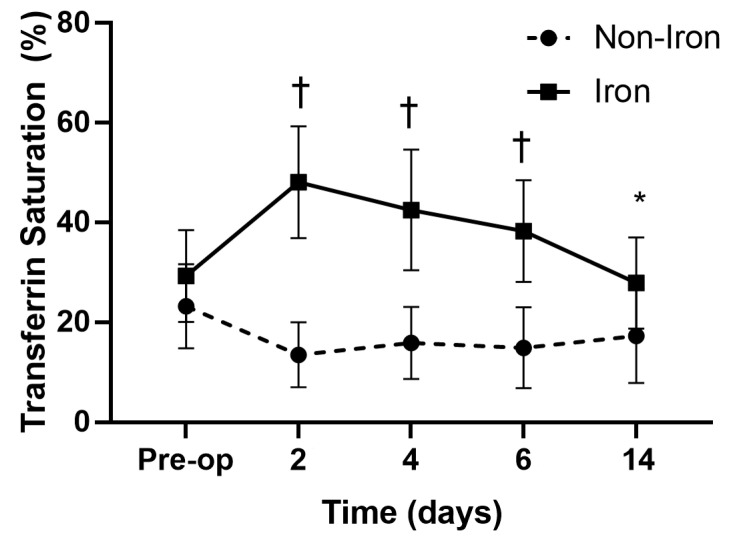
Serial changes in transferrin saturation. Transferrin values were significantly higher in the iron group than in the non-iron group on postoperative days 2, 4, 6, and 14. ^†^ *p* < 0.001, * *p* = 0.012.

**Figure 5:**
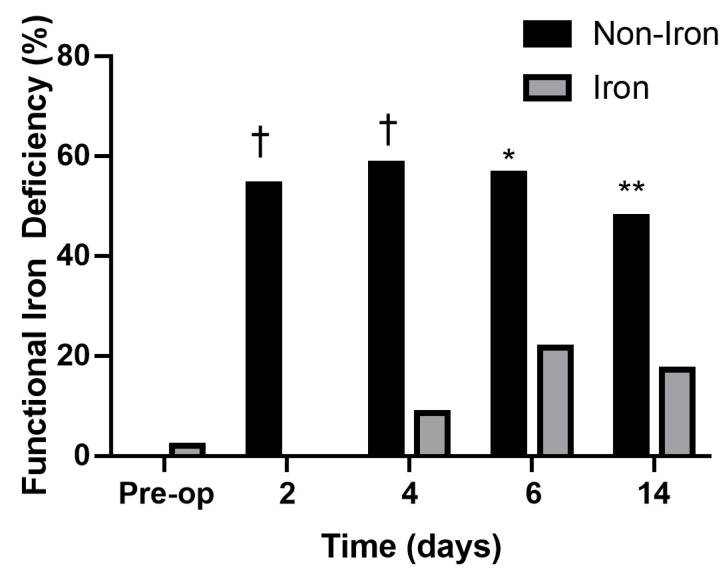
Serial changes in functional iron deficiency (%). Functional iron deficiency values were significantly lower in the iron group than in the non-iron group on postoperative days 2, 4, 6, and 14. ^†^ *p* < 0.001, * *p* = 0.001, ** *p* = 0.002.

Age, sex, BMI, and ASA-PS scores did not differ between the groups (Table 1). The iron group had significantly higher Hb levels on PODs 6 (10.5 ± 1.3 vs. 9.8 ± 0.9 g/dL, *p* = 0.032), 14 (11.3 ± 1.2 vs. 10.4 ± 1.1 g/dL, *p* = 0.021), and 30 (12.8 ± 1.1 vs. 11.9 ± 1.1 g/dL, *p* = 0.019) than the non-iron group (Figure 2). Additionally, the iron group had higher ferritin levels and TSATs on PODs 2, 4, 6, and 14 than the non-iron group did (Figure 3 and Figure 4). The rate of absolute iron deficiency was not different between the two groups preoperatively (2.1% vs. 4.2%, *p* = 0.210). Additionally, absolute iron deficiency was not observed in either group after surgery. However, the rate of functional iron deficiency was significantly higher in the non-iron group than in the iron group on PODs 2 (54.3% vs. 0%, *p* < 0.001), 4 (58.5% vs. 8.6%, *p* < 0.001), 6 (56.5% vs. 21.7%, *p* = 0.001), and 14 (47.8% vs. 17.3%, *p* = 0.002) (Figure 5). The CRP values were not different between the two groups on PODs 2 (39.7 mg/L vs. 41.5 mg/L, *p* = 0.411), 4 (61.7 mg/L vs. 63.2 mg/L, *p* = 0.603), 6 (43.7 mg/L vs. 41.2 mg/L, *p* = 0.432), and 14 (6.8 mg/L vs. 7.2 mg/L, *p* = 0.729). The rate of transfusion did not differ between the two groups (non-iron vs. iron group: 10.8% vs. 6.5%, *p* = 0.238). However, LOS was significantly shorter in the iron group than in the non-iron group (7.5 ± 1.6 days vs. 8.4 ± 2.3 days, *p* = 0.030). There were no postoperative complications in either group up to POD 30.

## 4. Discussion

The primary findings of this study were as follows: (1) the iron group had faster Hb recovery at PODs 6, 14, and 30; (2) the iron group had a significantly lower rate of functional IDA during the study period; (3) the rate of transfusion was not different between the iron and non-iron groups; and (4) LOS was shorter in the iron group than in the non-iron group. To the best of our knowledge, this is the first clinical study to analyze postoperative anemia and functional recovery following IV iron administration one day before TKA.

Conventionally, oral iron has been used to treat IDA [17]. However, the efficacy of oral iron is compromised because of poor absorption, poor compliance, and gastrointestinal side effects [8,18,19]. Das et al. [7] found that IV iron sucrose administration had a better safety and efficacy profile in the treatment of IDA than conventional oral iron supplements. In addition, Macdougall et al. [8] found that, among oral, intramuscular, and IV iron administration methods, IV was the most reliable method for reducing adverse risks and optimizing erythropoietin response. Deloughery et al. [20] found that oral iron administration was often poorly tolerated and that up to 70% of patients in their study complained of gastrointestinal issues. According to a recent meta-analysis of 20,000 patients from 103 studies, IV iron administration can effectively treat anemia without increasing adverse events or the incidence of infection when compared to oral or intramuscular iron administration [21]. Therefore, IV administration is considered more effective than oral or intramuscular administration.

Currently, three IV iron formulations (iron sucrose, ferric carboxymaltose, and ferric derisomaltose) are most commonly used because they do not have severe side effects [22]. Many studies have compared the efficacy and safety of different IV iron formulations [23,24,25,26]. Derman et al. [23] analyzed IV ferric derisomaltose and IV iron sucrose administration and found that the former required fewer administrations and showed a higher and faster Hb response than the latter. Wolf et al. [26] compared IV iron carboxymaltose and IV ferric derisomaltose and found that IV ferric derisomaltose administration led to a lower incidence of hypophosphatemia over 35 days, and that it was a safer treatment for anemia than IV iron carboxymaltose administration. In addition, Pollock et al. [25] found that IV ferric derisomaltose was more effective, even when used less frequently, than IV iron carboxymaltose, and thus was more cost-effective. Therefore, when comparing several studies, IV ferric derisomaltose has been regarded as an effective and safe formulation.

The current protocol states that IV iron should be administered to IDA patients 4 weeks before surgery; although this is the ideal treatment, it is not always possible in the field of orthopedic surgery [4,27]. Jeong et al. [4] indicated that this lack of feasibility was due to time constraints, requiring frequent outpatient visits and multiple blood sampling tests. Moreover, several reports indicate that IV iron administration used in the perioperative period was effective for treating postoperative anemia after TKA [10,28,29]. Yoo et al. [10] found that intraoperative IV ferric derisomaltose administration was an effective treatment for recovery from postoperative anemia after TKA. Similarly, Park et al. [5] found that intraoperative IV iron carboxymaltose administration was effective in the treatment of postoperative anemia in patients who had undergone TKA. However, to date, a limited number of studies have analyzed the effect of preoperative administration of IV iron one day before TKA. IV iron administration one day before TKA may have several advantages when compared with intraoperative administration, considering that it can be administered in a hemodynamically stable state, and as there are no interactions with other drugs [30,31,32].

In this study, serum ferritin and TSAT were higher and the rate of functional IDA was lower (during the study period) in the iron group than in the non-iron group, which resulted in faster Hb recovery of patients in the iron group. Serum ferritin levels reflect the total amount of iron stored in the body, and TSAT reflects the amount of circulating iron, which can be directly used in erythropoiesis [33,34,35]. Several studies have shown that circulating iron levels were more crucial to postoperative anemia recovery than total iron levels [36,37,38]. Functional IDA can be caused by the TKA-induced inflammatory response, in which the available quantities of total iron are sufficient, but iron mobilization is decreased due to an increase in hepcidin synthesis. Hepcidin inhibits ferroportin (an iron transporter), which regulates iron release from the body’s storage reserves to the bloodstream for erythropoiesis. After TKA, circulating iron is insufficient for anemia recovery owing to acute blood loss and decreased iron mobilization due to TKA-induced inflammation. In the iron group, IV iron administration quickly restored iron utilization to prevent the development of functional iron deficiency and promoted the erythropoietic response to quickly recover from anemia. In the non-iron group, insufficient circulating iron due to iron sequestration from blood loss hindered recovery from anemia [19,39,40]. Even though the rate of functional iron deficiency was significantly lower in the iron group than in the non-iron group on PODs 2, 4, 6, 14, the rate of functional iron deficiency in the iron group increased over time. Additional studies should investigate the optimal dose of iron supplementation required to further reduce the rate functional iron deficiency.

In our study, despite faster Hb recovery and shorter LOS in the iron group, the transfusion rate was not different between the two groups. Regarding LOS, several studies have already shown that postoperative anemia was associated with postoperative functional recovery after major orthopedic surgery [41,42,43]. Foss et al. [41] found that the degree of anemia was associated with functional mobility in the early postoperative period. Moreover, Lawrence et al. [42] found that patients with higher postoperative Hb levels had higher functional recovery. We agree that LOS does not reflect all aspects of functional recovery after TKA. However, considering that the same surgical certain duration of time technique and rehabilitation program were performed, and that consistent discharge criteria were strictly applied to all patients, we believe that LOS could be a useful indicator of functional recovery. Regarding the transfusion rate, contrary to our study findings, the results of several reports show that IV iron administration could lower the risk of transfusion [44,45,46,47]. However, postoperative transfusion is determined by the amount of acute blood loss that occurs during surgery; therefore, most transfusions occur within 1–2 days (hyper-acute phase) after surgery. We assumed that the effect of IV iron administration one day before surgery has not been demonstrated yet within the hyper-acute phase. In addition, it may be possible that our IV iron dose and administration timing were not optimal to achieve a low transfusion rate. Considering that several authors have reported that the transfusion rate was different according to the IV iron dose and administration timing [48,49], the optimal dose and timing of iron supplementation to reduce transfusion rates in TKA should be investigated in the future.

Our study has some limitations. First, this study had a relatively short-term follow-up period. Additional studies with long-term follow-up are warranted to evaluate the changes in Hb, ferritin, and TSAT levels and to identify whether the differences in Hb and iron profile levels between the two groups can lead to differences in patient-reported outcomes in the long term. Second, due to the retrospective design of the study, there may be concern regarding possible selection bias. However, the treatment decision of whether to administer IV iron was based on the date of the surgery, not on other parameters. Furthermore, patients in both groups were treated using the same surgical procedure with identical perioperative management. Moreover, propensity score matching was also performed to appropriately set the usage of IV iron as the only independent variable. Thirdly, we assumed that rapid Hb recovery affects patients’ functional recovery and leads to a shorter hospital stay. It should be noted that various factors, other than Hb level, could potentially affect postoperative functional recovery or LOS. Given the retrospective design of the study, other confounding factors may not have been controlled, and could have influenced the results. Lastly, this study was performed using a relatively small sample size due to the limited number of eligible patients. Prospective studies with a sufficient power analysis are warranted to confirm our findings.

## 5. Conclusions

IV iron administered one day prior to TKA was associated with postoperative anemia recovery and LOS, although it did not lower the postoperative transfusion rate. As approximately half of the patients undergoing TKA experience postoperative functional IDA, clinicians may consider the use of IV iron to improve iron availability. Furthermore, based on our study, further studies are warranted to investigate the definite relationship between IV iron administered one day prior to TKA and postoperative functional recovery.

## Figures and Tables

**Table 1:** Preoperative data after propensity score matching.

	**Non-Iron Group (n = 46)**	**Iron Group (n = 46)**	***p*** **-Value**
Age (years)	68.2 ± 6.5	69.1 ± 5.5	0.720
Males:females	8:38	6:40	0.562
BMI (kg/m^2^)	25.2 ± 3.2	25.4 ± 3.5	0.716
ASA-PS classification	2.2 ± 0.4	2.1 ± 0.3	0.587

ASA-PS, American Society of Anesthesiologists Physical Status; BMI, body mass index.

## Data Availability

Data presented in this study are available on request from the corresponding author. Data are not publicly available.
